# Mustercurriculum der Deutschen Gesellschaft für Rheumatologie für die Weiterbildung im Fachgebiet Innere Medizin und Rheumatologie

**DOI:** 10.1007/s00393-021-01053-9

**Published:** 2021-07-23

**Authors:** Alexander Pfeil, Martin Krusche, Diana Vossen, Michael N. Berliner, Gernot Keyßer, Andreas Krause, Hanns-Martin Lorenz, Bernhard Manger, Florian Schuch, Christof Specker, Jürgen Wollenhaupt, Xenofon Baraliakos, Martin Fleck, Fabian Proft

**Affiliations:** 1 Klinik für Innere Medizin III, Funktionsbereich Rheumatologie, Universitätsklinikum Jena, Am Klinikum 1, 07747 Jena, Deutschland; 2 Medizinische Klinik mit Schwerpunkt Rheumatologie und Klinische Immunologie, Charité Universitätsmedizin, Berlin, Deutschland; 3 Rheinisches Rheumazentrum Meerbusch-Lank, St. Elisabeth Hospital, Meerbusch, Deutschland; 4 Rheumatologie und Geriatrie, Helios Klinikum Berlin-Buch, Berlin, Deutschland; 5 Department für Innere Medizin, Klinik für Innere Medizin II, Universitätsklinikum Halle, Halle (Saale), Deutschland; 6 Klinik für Innere Medizin, Abteilung Rheumatologie, klinische Immunologie und Osteologie, Immanuel Krankenhaus Berlin, Berlin, Deutschland; 7 Sektion Rheumatologie, Medizinische Klinik V, Universitätsklinikum Heidelberg, Heidelberg, Deutschland; 8 Medizinische Klinik 3, Rheumatologie und Immunologie, Universitätsklinikum Erlangen, Friedrich-Alexander Universität Erlangen-Nürnberg, Erlangen, Deutschland; 9 Internistische Praxisgemeinschaft Rheumatologie – Nephrologie, Erlangen, Deutschland; 10 Klinik für Rheumatologie und Klinische Immunologie, Evangelisches Krankenhaus Kliniken Essen-Mitte, Essen, Deutschland; 11 Rheumatologie im Struenseehaus, Hamburg, Deutschland; 12 Rheumazentrum Ruhrgebiet, Ruhr-Universität Bochum, Herne, Deutschland; 13 Klinik und Poliklinik für Innere Medizin I, Universitätsklinikum Regensburg, Regenburg, Deutschland; 14 Klinik und Poliklinik für Rheumatologie/Klinische Immunologie, Asklepios Klinikum Bad Abbach, Bad Abbach, Deutschland; 15 Abteilung für Rheumatologie, Medizinische Klinik für Gastroenterologie, Infektiologie und Rheumatologie, Campus Benjamin Franklin, Charité Universitätsmedizin, Berlin, Deutschland

**Keywords:** Musterweiterbildungsordnung, Kernkompetenzen, Facharztweiterbildung, Ausbildungsabschnitte, Weiterbildungsort, Model advanced training regulation, Core competence, Medical specialist training, Training stage, Advanced training location

## Einleitung

Mit der Implementierung der Musterweiterbildungsordnung (MWBO) 2018 erfolgten die Anpassung der Gebietsdefinition sowie die Neustrukturierung der Weiterbildungszeit für den Facharzt bzw. die Fachärztin für Innere Medizin und Rheumatologie [2].

Die Mindestweiterbildungszeit umfasst hierfür 72 Monate. Hierbei wird die Weiterbildungszeit in 2 Teile zu je 36 Monaten aufgeteilt, welche auf der einen Seite die gemeinsamen Inhalte der Facharztweiterbildungen im Gebiet der Inneren Medizin („Basisweiterbildung“) als auch die spezifischen Inhalte der Facharztweiterbildung Innere Medizin und Rheumatologie umfasst.

Im allgemeinen Teil „Innere Medizin“ erfolgt der Erwerb von mindestens 2 Facharztkompetenzen, die nicht dem Schwerpunkt angehören, über 24 Monate sowie jeweils eine 6‑monatige Tätigkeit in einer Notaufnahme und der Intensivmedizin. Die spezialisierte Weiterbildungszeit im Fachgebiet Innere Medizin und Rheumatologie erstreckt sich ebenfalls über 36 Monate, von denen nach der MWBO mindestens 24 Monate in der stationären rheumatologischen Versorgung vorgesehen sind [2]. Von dieser Aufteilung abweichend, wurden in einzelnen Landesärztekammern Änderungen vorgenommen, die eine längere Weiterbildungszeit in der ambulanten rheumatologischen Versorgung ermöglichen.

Basierend auf der MWBO von 2018, wurde von der Kommission für Fort- und Weiterbildung der Deutschen Gesellschaft für Rheumatologie (DGRh) im Auftrag des Vorstandes der DGRh ein Mustercurriculum hinsichtlich der Kernkompetenzen in der Weiterbildung im Fachgebiet Innere Medizin und Rheumatologie entwickelt. Dieses Mustercurriculum fokussiert auf die Weiterbildungskompetenzen im Schwerpunkt während der 36 Monate Weiterbildungszeit im Fachgebiet Innere Medizin und Rheumatologie und beinhaltet keine Weiterbildungskompetenzen für den allgemeinen Teil der Inneren Medizin („Basisweiterbildung“).

Im Rahmen dieses Übersichtsartikels soll das Mustercurriculum der Deutschen Gesellschaft für Rheumatologie für die Weiterbildung im Fachgebiet Innere Medizin und Rheumatologie vorgestellt werden.

## Die Musterweiterbildungsordnung (MWBO)

In der MWBO 2018 werden „kognitive und Methodenkompetenzen“ (Kenntnisse) und „Handlungskompetenzen“ (Erfahrungen und Fertigkeiten) für das Fachgebiet Innere Medizin und Rheumatologie vermittelt [1, 2], welche die Erkennung, Diagnosestellung, Differenzialdiagnose, Therapie, Langzeitbetreuung und Rehabilitation entzündlich-rheumatischer Gelenkerkrankungen, inflammatorischer bzw. immunologischer Systemerkrankungen (insbesondere Kollagenosen und Vaskulitiden), autoinflammatorischer Syndrome, Immundefekte und der Komorbiditäten beinhalten [2]. Angelehnt an die MWBO 2018 erfolgte die Entwicklung des Mustercurriculums durch die Kommission Fort- und Weiterbildung der Deutschen Gesellschaft für Rheumatologie.

## Das Mustercurriculum

Ziel des Mustercurriculums ist die Etablierung eines Leitfadens für Weiterbilderinnen und Weiterbilder sowie Weiterzubildende in der Rheumatologie, in dem die Weiterbildungskompetenzen der 36-monatigen Weiterbildungszeit im Fachgebiet Innere Medizin und Rheumatologie, basierend auf der MWBO 2018, strukturiert und zeitlich abgestimmt vermittelt werden [2]. Das Mustercurriculum soll auch als Grundlage für alle Weiterbilderinnen und Weiterbilder bei der Ausarbeitung eines eigenen Weiterbildungsplanes dienen, der mit der Umsetzung der MWBO bei den Landesärztekammern bei der erforderlichen Neubeantragung der Weiterbildungsbefugnis eingereicht werden muss.

Als Grundlage für das Mustercurriculum dienen die in der MWBO 2018 aufgeführten definierten Untersuchungs- und Behandlungsverfahren [2]. Ziel des Mustercurriculums ist das strukturierte Erlernen der körperlichen Untersuchung, Diagnose, Differenzialdiagnose, Therapiealgorithmen, Langzeitbetreuung und Rehabilitation entzündlich-rheumatischer Gelenkerkrankungen, inflammatorischer bzw. immunologischer Systemerkrankungen (insbesondere Kollagenosen, Vaskulitiden und autoinflammatorische Syndrome) sowie deren assoziierten Komorbiditäten.

## Aufbau des Mustercurriculums

Die zu vermittelnden und zu erlernenden Kompetenzen wurden auf 3 Weiterbildungsjahre aufgeteilt. Bei den Kompetenzen handelt es sich um Mindestanforderungen, welche in Adaptation an die jeweilige Weiterbildungsstätte angepasst werden sollten, sodass entsprechende Tätigkeiten oder Kenntnisse zu einem früheren (aber auch späteren) Zeitpunkt erlernt oder durchgeführt werden können. Selbstverständlich ist eine vertiefte Kompetenzvermittlung über das vorliegende Mustercurriculum hinaus möglich und wünschenswert und kann entsprechend im individuellen Weiterbildungsplan der Weiterbildungsstätte aufgenommen werden. Die hier vorgenommene Strukturierung der Weiterbildungskompetenzen wurde in der Kommission für Fort- und Weiterbildung erarbeitet und vom Vorstand der DGRh konsentiert und soll einen zeitlich abgestimmten Kompetenzerwerb ermöglichen. Additiv werden variable Weiterbildungsinhalte abgebildet, welche keine Zuordnung zum Weiterbildungsabschnitt erfahren und entsprechend dem Kenntnisstand im ersten bis dritten Weiterbildungsjahr vermittelt werden können.

Der Kompetenzerwerb folgt einer Gliederung des Curriculums hinsichtlich des Erlernens von Leitsymptomen, Untersuchung, Labor, Radiologie, rheumatologischer Diagnostik, physikalischer Therapie und therapeutischen Maßnahmen. Zusätzlich erfolgt im zweiten und dritten Weiterbildungsjahr das Erlernen von Interventionen (z. B. Gelenkpunktion) (*Details *s. Tab. 1).

**Table 1:** 

	Kompetenzerwerb
**1. Jahr**	*Leitsymptom:* Erlernen einer gezielten rheumatologischen Anamnese mit Fokussierung auf das führende Leitsymptom und hierfür möglicherweise zugrunde liegende entzündlich-rheumatische Erkrankungen
	*Untersuchung:* Erlernen einer gezielten rheumatologischen körperlichen Untersuchung inklusive möglicher systemischer Beteiligungen entzündlich-rheumatischer Erkrankungen
	*Labor:* Erlernen einer gezielten Indikationsstellung sowie Einführung in Methodik, Durchführung und Einordnung von Laboruntersuchungen sowie immunologischer Parameter in die entzündlich-rheumatischen Krankheitsbilder
	*Radiologie:* Erlernen der gezielten Indikationsstellung radiologischer Untersuchungen sowie die Einordnung der Befunde in die entzündlich-rheumatischen Krankheitsbilder
	*Rheumatologische Diagnostik:* Einführung in gezielte rheumatologisch-immunologische Diagnostik mittels Arthro- und Gefäßsonographie, Kapillarmikroskopie, Analyse der Synovialflüssigkeit sowie rheumatologische/immunologische Labordiagnostik einschließlich Autoantikörper und immungenetische Testung unter engmaschiger Anleitung
	*Physikalische Therapie:* Kenntnisse physikalischer, krankengymnastischer und ergotherapeutischer Behandlungsprinzipien spezifischer entzündlich-rheumatischer Erkrankungen und degenerativer Gelenkerkrankungen sowie Wirbelsäulenerkrankungen
	*Therapie:* – Kenntnisse über die Erstellung eines medikamentösen Therapieplans zur Behandlung entzündlich-rheumatischer Erkrankungen und degenerativer Gelenkerkrankungen – Erlernen des Erkennens von – und den Umgang mit – möglichen therapieassoziierten unerwünschten Ereignissen – Selbstständige Verordnung medikamentöser Therapien sowie von Hilfsmitteln anhand der Heilmittelverordnung zur Behandlung entzündlich-rheumatischer Erkrankungen und degenerativer Gelenkerkrankungen – Erlernen der interdisziplinären Indikationsstellung zu chirurgischen, strahlentherapeutischen und nuklearmedizinischen Behandlungsverfahren – Mitbetreuung von Patienten mit entzündlich-rheumatischen Erkrankungen und degenerativen Gelenkerkrankungen unter Supervision – Erstellen strukturierter rheumatologischer Befunde/Arztbriefe unter Supervision
**2. Jahr**	*Leitsymptom/Diagnose/Differenzialdiagnose:* Vertiefung der gezielten rheumatologischen Anamnese zur differenzialdiagnostischen Einordnung der geschilderten Beschwerdesymptomatik
	*Untersuchung:* Vertiefung der gezielten rheumatologischen körperlichen Untersuchung inklusive möglicher systemischer Beteiligungen entzündlich-rheumatischer Erkrankungen
	Selbstständige Betreuung von Patienten mit entzündlich-rheumatischen Erkrankungen und degenerativen Gelenkerkrankungen unter Supervision
	*Radiologie:* vertiefende Kenntnisse der Auswertung radiologischer Verfahren zur Diagnostik entzündlich-rheumatischer und degenerativer Gelenkerkrankungen sowie möglicher therapieassoziierter unerwünschter Ereignisse und die differenzialdiagnostische Einordnung dieser Befunde in die entzündlich-rheumatischen Krankheitsbilder
	*Rheumatologische Diagnostik:* selbstständige Durchführung gezielter rheumatologisch-immunologischer Diagnostik mittels Arthrosonographie, Gefäßsonographie, Kapillarmikroskopie, Analyse der Synovialflüssigkeit sowie rheumatologische/immunologische Labordiagnostik einschließlich Autoantikörper, zelluläre Immunität und immungenetische Testung unter Supervision
	*Interventionen:* Einführung in invasive diagnostische und therapeutische Verfahren (wie z. B. Gelenkpunktion, Injektionstherapien und/oder Lippendrüsen- und Hautbiopsien) unter engmaschiger Anleitung
	*Therapie:* selbstständige Erstellung eines medikamentösen Therapieplans zur Behandlung entzündlich-rheumatischer Erkrankungen und degenerativer Gelenkerkrankungen inklusive Aufklärung der Patientinnen/Patienten unter Supervision
**3. Jahr**	*Anamnese, Diagnostik, Therapie:* selbstständige Betreuung von Patienten mit entzündlich-rheumatischen Erkrankungen und degenerativen Gelenkerkrankungen mit der Möglichkeit zur Rücksprache bei Bedarf
	Erwerb von Grundkenntnissen in physikalischer und rehabilitativer Medizin sowie zu Fragestellungen der rheumatologischen Begutachtung
	Erwerb von Kenntnissen zur Transition von Patienten mit pädiatrisch-rheumatologischen Krankheitsbildern
	Erwerb von Grundkenntnissen zur Planung und Betreuung von Schwangerschaften bei Patientinnen mit entzündlich-rheumatischen Erkrankungen
	Erwerb von Grundkenntnissen zur Diagnostik und Therapie von primären und sekundären Immundefekten
	*Radiologie:* Auswertung radiologischer Verfahren zur Diagnostik entzündlich-rheumatischer und degenerativer Gelenkerkrankungen sowie möglicher therapieassoziierter unerwünschter Ereignisse und die differenzialdiagnostische Einordnung dieser Befunde in die entzündlich-rheumatischen Krankheitsbilder
	*Rheumatologische Diagnostik:* selbstständige Durchführung gezielter rheumatologisch immunologischer Diagnostik mittels Arthrosonographie, Gefäßsonographie (inklusive Duplexsonographie zur Akutdiagnostik der Vaskulitiden), Ultraschall der Speicheldrüsen, Kapillarmikroskopie, Analyse der Synovialflüssigkeit sowie rheumatologische/immunologische Labordiagnostik einschließlich Autoantikörper und immungenetische Testung mit der Möglichkeit zur Rücksprache bei Bedarf sowie Erlernen der Qualitätssicherung gemäß RiLiBÄK-Richtlinien
	*Interventionen:* Durchführung invasiver diagnostischer und therapeutischer Verfahren (wie z. B. Gelenkpunktion und Injektionstherapien und anderen) unter Supervision

Als variable Inhalte, welche keinem Weiterbildungsabschnitt zugeordnet werden, sind die Osteologie, Schulungsprogramme und Themen des Strahlenschutzes zu nennen (s. Tab. 2).

**Table 2:** 

	Kompetenzerwerb
**Zeitlich variable Zuordnung**	*Osteologie:* – Diagnostik und konservative Therapie der Osteoporose und Osteomalazie bzw. osteologischer Erkrankungsbilder – Indikationsstellung, Durchführung und Auswertung der DXA-Knochenmineraldichtemessung
	*Schulungsprogramme:* Kennenlernen und Erlernen von strukturierten Patientenschulungsprogrammen bei rheumatischen und muskuloskeletalen Erkrankungen
	*Strahlenschutz:* – Grundlagen der Strahlenbiologie und Strahlenphysik bei der Anwendung ionisierender Strahlen am Menschen – Grundlagen des Strahlenschutzes beim Patienten und Personal einschließlich der Personalüberwachung und des baulichen und apparativen Strahlenschutzes

## Anpassung des Mustercurriculums

Das Mustercurriculum reflektiert die Mindestanforderungen der MWBO. In diesem Zusammenhang ist das Mustercurriculum nicht als optimale und bindende Weiterbildungsvariante für jede rheumatologische Weiterbildungsstätte in Deutschland anzusehen, sondern gibt den Rahmen für eine erfolgreiche Weiterbildung zum/zur Facharzt/Fachärztin für Innere Medizin und Rheumatologie vor. Eine Anpassung des Curriculums an die jeweiligen Gegebenheiten des Weiterbildungsstandortes ist jederzeit möglich bzw. ggf. notwendig. Dies beinhaltet Änderungen der zeitlichen Abfolge der Wissensvermittlung, eine vertiefte Weiterbildung in einzelnen Aspekten sowie die Hinzunahme von weiteren Weiterbildungsinhalten.

Für eine kontinuierliche Weiterentwicklung des Curriculums benötigt die Kommission für Fort- und Weiterbildung eine Rückmeldung der Weiterbilderinnen und Weiterbilder sowie der Weiterzubildenden hinsichtlich der Weiterbildungsinhalte, -themen und Struktur des Curriculums. Somit ist das Curriculum als „Work in Progress“ zu sehen, weshalb ausdrücklich Kommentare oder auch Verbesserungsvorschläge an die Kommission erwünscht sind.

Zusammenfassend soll durch das Mustercurriculum eine standardisierte und strukturierte Aus- und Weiterbildung von Fachärztinnen und Fachärzten für Innere Medizin und Rheumatologie gewährleistet werden.

## Fazit für die Praxis

- Das Mustercurriculum ermöglicht die standardisierte Vermittlung von Kernkompetenzen im Rahmen der Facharztweiterbildung für Innere Medizin und Rheumatologie.
- Die zu erlernenden Kernkompetenzen und die variabel zuzuordnenden Weiterbildungsinhalte werden in 3 Ausbildungsabschnitten über 36 Monate vermittelt.
- Eine Anpassung des Curriculums an die Gegebenheiten des jeweiligen Weiterbildungsortes ist sinnvoll bzw. notwendig.

## Abkürzungen

DGRh: Deutsche Gesellschaft für Rheumatologie
MWBO: Musterweiterbildungsordnung
